# The Impact of Gender Diversity on Excellence in Pathology Research and Education

**DOI:** 10.7759/cureus.56114

**Published:** 2024-03-13

**Authors:** NFN Kiran, Pooja Devi, Meena Kashi, FNU Anjali, Saroja Devi Geetha

**Affiliations:** 1 Pathology, Staten Island University Hospital, New York City, USA; 2 Pathology/Hematopathology, University of Pennsylvania Health System, Philadelphia, USA; 3 Pathology, Northwell Health, New York City, USA; 4 Internal Medicine, Sakhi Baba General Hospital, Sukkur, PAK; 5 Pathology, Zucker School of Medicine, North Shore University Hospital/Long Island Jewish Medical Center, Northwell Health, Greenvale, USA

**Keywords:** innovations, gender parity, barriers in pathology, pathology, gender disparity

## Abstract

In this editorial, we inspect the critical role of gender diversity within the domain of pathology and its consequential impact on research innovation and clinical outcomes. The editorial commences with a historical overview of gender disparities in pathology, acknowledging advancements toward gender parity while highlighting persistent impediments to full inclusivity. The discourse emphasizes the intrinsic value of integrating diverse gender perspectives in research, illustrating how such inclusivity catalyzes innovation, mitigates research biases, and elevates the standard of patient care through a more comprehensive understanding of the field of pathology. Key barriers to gender diversity in pathology are systematically analysed, including disproportionate clinical burdens, time allocation conflicts due to societal roles, restricted access to specialized training, financial limitations, inadequate support networks, workplace discrimination, and the challenge of balancing family responsibilities with professional aspirations. We propose strategic interventions to address these barriers, advocating for increased awareness, diversity-focused training programs, and mechanisms for recognizing and rewarding the contributions of underrepresented genders in the field.

Furthermore, we highlight exemplary initiatives that have successfully promoted gender diversity, such as the Johns Hopkins Pathology Department's outreach program, and the role of professional organizations, notably the American Society for Investigative Pathology and its "Women in Pathology" community, is discussed as pivotal in celebrating and advancing women's contributions to the field of pathology.

In conclusion, we suggest that dismantling gender bias is imperative for realizing the full potential of pathology as a discipline. The editorial argues for a systemic embrace of gender diversity and inclusivity as fundamental to fostering research innovation, enhancing clinical practice, and ultimately improving patient outcomes. This scholarly examination calls for a concerted effort within the pathology community to integrate diverse perspectives, thereby enriching the field and contributing to the advancement of medical science.

## Editorial

Introduction

Over the past 20 years, the intersection of pathology and research has become fundamental to the advancement of medical science. Pathology, in particular, has emerged as a cornerstone in diagnosis, research, and medical education, highlighting the indispensable role of pathologists in introducing and validating new findings and interventions within clinical practice. This synergy between pathology specialty and research is crucial, allowing for the seamless integration of novel interventions into healthcare settings, thereby minimizing potential risks associated with unverified practices.

Highlighting systematic discrepancies

Historically, the field of pathology, like many other medical disciplines, was predominantly male, a reflection of broader gender biases and barriers within academia and society [1]. These barriers ranged from entrenched gender stereotypes to limited opportunities and the disproportionate burden of domestic responsibilities shouldered by women. According to a recent survey, the United States and Europe are suffering from a national health crisis, as well as discrepancies in the quality and frequency with which racial and ethnic minorities receive treatment [2]. Almost half of the medical school applicants worldwide are female; however, only 39% of them are currently affiliated with an academic medicine as faculty [3]. Nevertheless, the landscape of the field of pathology has undergone significant transformation in recent years, as evidenced by a shift towards more inclusive gender and racial representation in relation to the leading authors of high impact medical studies and randomized controlled trials [4,5]. These efforts aimed at promoting gender equality and dismantling longstanding biases have begun to reshape the academic community, though much work remains to be done to achieve true equality.

According to 2022 demographics, 78.9% of all attending pathologists were men, whereas women contributed only 21.1% to the total workforce. It was further investigated that women, on average, earn 84¢ for every $1 earned by men [6]. A subsequent study conducted in 2021 yielded remarkably comparable findings. Among 5228 individuals, 57% were male and 40% were female academic pathologists [7]. Notably, within this group, males occupied a disproportionately higher number of senior academic ranks and leadership positions. The underrepresentation of women demands extensive discussion since it has long-term detrimental impacts on the field. For instance, it restricts an array of perspectives, impeding creativity and problem-solving. Furthermore, it reinforces prejudices and biases in research and patient care. Lack of gender diversity also deprives the field of talented individuals, limiting its potential for growth and brilliance. Such disparities underscore the need for continued efforts to foster gender diversity in pathology.

Advantages of embracing diversity in research

The integration of diverse perspectives into research is not just beneficial, but also essential for fostering innovation and excellence in today's rapidly evolving scientific landscape. The collaborative synergy achieved through gender diversity in research teams unleashes innovative strategies, as it draws upon the rich tapestry of experiences, backgrounds, ages, genders, and expertise. This amalgamation of perspectives propels the field forward, making significant strides in novel research methodologies and findings.

Tackling With Bias

One of the most critical outcomes of this diversity is the substantial reduction in bias. Traditional research approaches, often constrained by a homogeneity of thought, are prone to biases that can skew results and interpretations. The inclusion of varied viewpoints effectively dilutes these biases, offering a more balanced and comprehensive understanding of research subjects [8]. This multidimensional approach not only enriches the research process but also paves the way to excellence. Diversity, as a catalyst for excellence, drives advancement and innovation, particularly vital for addressing the complex health needs of diverse populations.

Enhancing Patient Care

The role of diversity in improving patient care cannot be overstated. A broader understanding of pathological processes, diagnostic procedures, treatments, and prognostic outcomes is achievable through the lens of diverse research teams. Such inclusivity leads to superior healthcare outcomes, as diverse medical staff are inherently more adept at catering to the nuanced needs of a varied patient demographic [8].

Empowering Leadership and Promoting Mentorship

The ripple effects of gender diversity extend into mentorship and leadership within the medical and research communities. Overcoming gender biases not only facilitates increased representation of women in these fields but also encourages more women to ascend to leadership positions. This upward mobility serves as a beacon of inspiration for aspiring female researchers and pathologists, fostering a culture of mentorship where women can guide, support, and lead by example. The resultant shift towards greater equity not only enhances societal trust in the medical profession's impartiality but also strengthens the overall fabric of medical and research communities.

Economic Impact

The economic implications of diversity in research are profound. This is attributed to the accelerated pace of research, the development of lucrative fields, and the enhanced capacity for complex problem-solving [9]. Diverse research teams are more likely to gain funding and investment, create employment opportunities for adjacent businesses, and encourage an entrepreneurial culture, all of which contribute to economic growth and resilience.

Navigating the barriers: underrepresented genders in pathology research

The trajectory of careers in pathology research for individuals from underrepresented genders is fraught with systemic and societal challenges. These barriers, deeply rooted in gender stereotypes and structural inequities, significantly impede their progress in this demanding field.

Hegemonic Masculinity

The concept of "hegemonic masculinity" refers to a set of masculine characteristics, attitudes, and practices that are formed as the dominant and idealized norm and against which both men and women are judged [10]. It applies to the societal standards, attitudes, and behaviors associated with masculinity that are viewed as more attractive and powerful. It also acknowledges that hegemonic masculinity can exert influence and perpetuate power dynamics that disadvantage women. In pathology and research, the perpetuation of gendered power dynamics under the banner of hegemonic masculinity can lead to disparities in the distribution of resources and opportunity, further marginalizing women and gender-diverse individuals in the profession.

Heavy Clinical Load

There exists a pervasive stereotype that women are inherently less capable of managing the rigorous demands of healthcare environments. This misconception often discourages them from pursuing careers in specialised areas like pathological research, where the workload and expectations are high.

Time Constraints

Societal expectations disproportionately allocate the responsibilities of domestic duties and family care to women, limiting their available time to dedicate to their professional aspirations. Consequently, their career development often suffers due to these unbalanced expectations.

Insufficient Training Opportunities

The underrepresentation of certain genders in pathology research can also be traced back to a lack of accessible training and development opportunities. Addressing this gap requires targeted initiatives aimed at supporting and nurturing the talents of women and other underrepresented groups in the field.

Financial Constraints

Historically, men have been perceived to have greater financial autonomy to invest in their careers. In contrast, women have faced limitations due to less financial independence. While this scenario is gradually changing, the impact of financial constraints on career progression cannot be overlooked.

Workplace Inequities

Incidences of sexual harassment and workplace impropriety disproportionately affect female researchers, undermining their safety and comfort in professional settings. This issue highlights the need for stronger measures to ensure workplace safety and equity.

Family Responsibilities

Despite societal progress toward gender equality, the bulk of caregiving and domestic responsibilities still predominantly fall on women. This imbalance continues to challenge the notion of equal opportunities for all genders within professional domains.

Addressing these challenges requires a concerted effort from both within and outside the pathology research community. Instituting policies that support work-life balance, providing equitable training and development opportunities, ensuring financial support mechanisms, and fostering a safe and inclusive work environment are critical steps towards dismantling the barriers underrepresented genders face in pathology research.

Combating gender bias in educational environments

Recent studies revealed that 89% of female respondents reported encountering gender bias within their professional journeys, underscoring a pervasive issue that, while affecting all genders, disproportionately hinders women from advancing in their careers [1,8]. The European Policy of “Gender Mainstreaming” is important for the implementation of secure professional careers and for promoting gender diversity [11]. It has the following themes and approaches: equal treatment ensures that men and women receive equal treatment, constructive action involves making constructive efforts to address differences, formulating newer policies and strategic advancements that not only combat gender disparity but also allow economic growth across all genders, and implementing gender equality in systems, organizations, institutions, programs, policies, and practices. Cultivating such an environment is essential for a progressive, equitable workplace irrespective of one's chosen field.

Enhancing Inclusivity in Pathology Education

Addressing the barriers imposed by gender biases in pathology is not an insurmountable challenge. Even modest shifts in mindset, advocacy, and policy can catalyse a profound and lasting change, benefiting future generations. Several strategies can be pivotal in this transformation.

Raising awareness: Enlightening potential female pathologists about the opportunities within the field is crucial. Through seminars, and social and electronic media campaigns, we can underscore the importance of gender diversity, drawing more women into rewarding careers in pathology.

Implementing diversity training: Given the male dominance in leadership positions within pathology and research, targeted training programs are essential. These initiatives can educate and encourage male leaders to create safer, more welcoming environments for women. The influence of these leaders can be instrumental in reshaping societal perceptions and dismantling entrenched biases.

Recognition and incentivization: Instituting awards and recognitions serves as a powerful motivator, acknowledging exceptional talent and contributions to the field. This strategy not only honors individual achievements but also signals to prospective female pathologists the value and opportunities available within the discipline.

Additional Measures to Enhance Inclusivity

Increased exposure: Introducing women to the full spectrum of medical specialties, including those traditionally male-dominated, can broaden their career aspirations.

Accessibility: Streamlining access to educational and training programs ensures that all aspiring pathologists, regardless of gender, can pursue their professional goals without undue barriers.

Confronting implicit biases: Acknowledging and addressing the unconscious biases that pervade educational and professional settings is critical. By recognizing these biases, institutions can implement strategies to mitigate their impact, fostering a more inclusive and equitable environment. Through these concerted efforts, the field of pathology can move towards a future where gender inclusivity is not just an aspiration but a reality, enriching the discipline with diverse perspectives and innovations.

Pioneering gender diversity in pathology

The journey towards achieving gender diversity in pathology has seen the launch of several impactful initiatives, designed to counteract the gender biases pervading the field. Recognizing the underrepresentation of certain groups, the Johns Hopkins Pathology Department, in 2012, spearheaded a fully funded outreach program aimed at increasing participation [12]. This innovative program, encompassing faculty meetings and conferences, witnessed a remarkable increase in participants within a mere five-year span, signaling a significant stride towards inclusivity [4,5]. Similarly, the Diversity Initiative at the Wayne State University Eugene Applebaum College of Pharmacy and Health Sciences has broadened its focus beyond gender, embracing cultural diversity to reflect the multifaceted nature of society. These programs exemplify the proactive steps being taken to enrich the field of pathology with diverse voices and perspectives.

Advocating gender diversity through professional organizations

The establishment of the "Women in Pathology" community by the American Society for Investigative Pathology marks another milestone in the quest for gender diversity [13]. This initiative, led by esteemed female scientists, researchers, and pathologists from prestigious institutions, including the University of Chicago, Tufts University, and Brown University, serves as a platform for celebrating women's achievements in pathology. It also focuses on creating opportunities to enhance inclusivity and support for women within the field.

Conclusion

The presence of gender bias in pathology and research significantly hinders the advancement of these vital scientific fields. Addressing and dismantling this bias is crucial, though efforts to fully eradicate it are still ongoing. The pathology community must recognize and embrace the importance of gender diversity and inclusivity. By fostering an environment that values diverse perspectives, the field can enhance its contributions to healthcare. This includes leveraging varied research methodologies to drive innovation and improve patient outcomes. As the community moves forward, a shared commitment to these values will be key to creating a more equitable and effective future for pathology.
